# Poor expression of miR‐195‐5p can assist the diagnosis of cerebral vasospasm after subarachnoid hemorrhage and predict adverse outcomes

**DOI:** 10.1002/brb3.2766

**Published:** 2022-11-09

**Authors:** Yong Li, Senyuan Yang, Xiaobin Zhou, Runlong Lai

**Affiliations:** ^1^ Department of Neurosurgery The First Affiliated Hospital of Shantou University Medical College Shantou China

**Keywords:** adverse outcomes, cerebral vasospasm, diagnostic value, ET‐1, IL‐6, miR‐195‐5p, PDGF, subarachnoid hemorrhage

## Abstract

**Objective:**

Patients with spontaneous subarachnoid hemorrhage (SAH) may develop refractory arterial cerebral vasospasm (CVS), which is the leading cause of death in SAH patients. This study explored the clinical diagnostic value of serum miR‐195‐5p levels in CVS after SAH (SAH + CVS) and its relationship with the prognosis of SAH + CVS.

**Methods:**

A total of 110 patients with spontaneous SAH were divided into the SAH group (*N* = 62) and SAH + CVS group (*N* = 58), with 60 healthy subjects as controls. The clinical data of blood glucose, blood sodium fluctuation, and serum lactic acid were recorded. miR‐195‐5p serum level was detected by RT‐qPCR and its diagnostic value on SAH + CVS was analyzed by receiver operating characteristic curve. Serum levels of PDGF/IL‐6/ET‐1 and their correlation with miR‐195‐5p were analyzed using RT‐qPCR, enzyme‐linked immunosorbent assay, and Pearson's method. The patient prognosis was evaluated by Glasgow Outcome Scale. The effect of miR‐195‐5p levels on adverse prognosis was analyzed by Kaplan‐Meier method and Cox regression analysis.

**Results:**

miR‐195‐5p was lowly expressed in the serum of SAH patients and lower in SAH + CVS patients. Serum miR‐195‐5p level assisted the diagnosis of SAH and SAH + CVS and was negatively correlated with PDGF/IL‐6/ET‐1 levels and was an independent risk factor together with ET‐1 and blood glucose for SAH + CVS. miR‐195‐5p low expression predicted a higher cumulative incidence of adverse outcomes and was an independent predictor of adverse outcomes.

**Conclusion:**

Poor expression of miR‐195‐5p can assist the diagnosis of SAH + CVS and predict higher adverse outcomes.

## 1 INTRODUCTION

Subarachnoid hemorrhage (SAH) is a serious cerebrovascular disease by blood flowing into the subarachnoid space after intracranial vascular rupture (Shang et al., 2021). Depending on the etiology, it can be divided into traumatic and spontaneous SAH (Dubosh and Edlow, 2021). Intracranial aneurysm is the main cause of spontaneous SAH, accounting for about 85% of all cases (Neifert et al., 2021; Shang et al., 2021). Cerebral vasospasm (CVS) is a common complication of SAH, an important cause of disability and death caused by spontaneous SAH, and has always been considered one of the main threats to the poor prognosis after SAH (Etminan and Macdonald, 2021). Spasmogenic substances produced during subarachnoid wall dissolution and endothelial injury cause smooth muscle cell contraction, which is associated with the pathogenesis of CVS (Xu et al., 2021). Moreover, CVS after SAH may be caused by oxidative stress, inflammation, apoptosis, and toxic substances, but its specific pathophysiological mechanism is still unclear, and there is no effective treatment for CVS at present (Gulec et al., 2021; Haruma et al., 2016). Therefore, understanding the pathogenesis of CVS caused by SAH and its early detection and active treatment can save the patient's life and help judge the prognosis of the disease.

MicroRNAs (miRNAs) are noncoding single‐stranded RNAs, which are widely involved in gene transcription, cell proliferation, invasion, and angiogenesis (Bounds et al., 2017). Abnormal regulation of miRNAs is associated with a series of neurological diseases (Saugstad, 2010). For instance, miR‐15a was found to be a miRNA associated with SAH‐induced CVS and its expression was elevated in SAH patients (Kikkawa et al., 2017). It was reported that miR‐24 affects CVS caused by SAH by regulating NOS3 (Li et al., 2018). In addition, miR‐24 regulates inflammation and neurological function in rats with CVS after SAH by targeting HMOX1 (Deng et al., 2021). Moreover, miR‐195‐5p plays an important role in vasospasm induced by SAH in rats (Tsai et al., 2021). Yet, the effect of miR‐195‐5p on CVS after SAH has not been reported in clinical studies, so further verification is still needed.

Platelet‐derived growth factor (PDGF), a member of the growth factor family, has been widely concerned and reported to be involved in vasoconstriction (Zhang et al., 2010). In addition, PDGF can cause proliferation of cerebrovascular smooth muscle cells, apoptosis, and necrosis of vascular cells, leading to cerebrovascular wall remodeling and decreased vascular compliance, thus playing a fundamental role in CVS (Smits et al., 1991; van Heyningen et al., 2001). Borel et al. observed elevated PDGF‐β levels in human SAH patients, which may be related to peripheral thrombogenesis, and thus result in CVS (Cui et al., 2014). Nevertheless, the specific mechanism of its interaction with CVS caused by SAH is still unidentified. In addition, the clinical study on the role of miR‐195‐5p in CVS caused by SAH has not been reported at home and abroad. Accordingly, by detecting the serum expression of miR‐195‐5p in patients with SAH, the clinical significance of miR‐195‐5p on CVS was considered, providing certain reference value for the clinical application of CVS caused by SAH.

## 2 MATERIALS AND METHODS

### 2.1 Ethics statement

This study was approved by the Ethics Committee of The First Affiliated Hospital of Shantou University Medical College. Patients and their families were fully informed of the purpose of this study, and all voluntarily participated in this study and signed informed consent prior to sampling.

### 2.2 Study subjects

A total of 120 patients with spontaneous SAH hospitalized in the neurosurgery department of The First Affiliated Hospital of Shantou University Medical College from June 2015 to December 2020 were selected. According to the occurrence of CVS, they were allocated into the following two subgroups: patients with simple SAH (SAH group, *N* = 62) and patients with SAH and CVS (SAH + CVS group, *N* = 58). The diagnostic criteria for spontaneous SAH were as follows: cranial CT indicated increased density of sulci and cistern with high‐density shadow. Lumbar puncture and cerebrospinal fluid tests were performed on patients with negative or suspicious cranial CT scan results, and the results were consistent with SAH neuroimaging and cerebrospinal fluid signs, and trauma was excluded. Clinical manifestations and signs were as follows: (1) general brain circulation disorders, orientation, and attention disorders, progressive consciousness disorder, coma turned to consciousness and then turned to coma; (2) occurrence or aggravation of local cerebral circulation disorders, such as aphasia, unilateral paralysis, or hemiplegia; (3) increased intracranial pressure: after proper treatment of SAH, the increased intracranial pressure of patients was improved for a time, and the symptoms were aggravated when headache, vomiting, and papilledema occurred again; (4) the above symptoms cannot be explained by rebleeding or intracranial hematoma. The diagnostic criteria of CVS were that the mean velocity of middle cerebral artery measured by transcranial Doppler (TCD) is *Vm* > 120 cm/s. The clinical symptoms were as follows: deteriorative headache, neck rigidity, low fever, latent decreased level of consciousness, orientation disorder, and fluctuating focal neurological deficits occurred 5–14 days after SAH; rebleeding and hydrocephalus were excluded by brain CT scanning; other causes of neurological deterioration such as electrolyte disturbances, hypoxia, and seizures were excluded. In addition, 30 healthy people who underwent physical examinations in The First Affiliated Hospital of Shantou University Medical College during the same period were selected as the control group.

Inclusion criteria were as follows: diagnosed with spontaneous SAH; admitted to the hospital within 24 h after onset; without administration of anticoagulant drugs in the last 2 weeks; no history of head trauma in the last 3 weeks; cooperating with inspection and follow‐up.

Exclusion criteria were as follows: SAH secondary to intracranial infection, tumor stroke, and hematologic diseases; hyperlipidemia; diabetes history; dysfunction of vital organs such as heart, lung, liver, and kidney; diseases of the blood system and long‐term living in the plateau area; patients deemed unsuitable for clinical study by the investigator.

### 2.3 Sample and data collection

Upon admission, basic clinical information of all patients was collected, including age, gender, smoking history, drinking history, past medical history (hypertension, hyperlipidemia, diabetes), brain CT scanning results, blood glucose, blood sodium, and serum lactic acid. Fasting blood glucose, blood sodium, and serum lactic acid were rechecked and recorded within 7 days after admission. Similarly, relevant information about health examiners was collected during the physical examination. The fasting venous blood (5 mL) was collected in the morning and centrifuged after 30 min. Then, the serum was separated and stored at −80°C for measurement. The blood glucose level in the serum was monitored by Hitachi automatic biochemical analyzer; the concentration of serum sodium was detected with the ion electrode method; the level of serum lactic acid was determined with the oxidase method; the enzyme‐linked immunosorbent assay (ELISA) kit was employed to detect the serum levels of PDGF, interleukin (IL)−6, and endothelin‐1(ET‐1); reverse transcription quantitative polymerase chain reaction (RT‐qPCR) was conducted to detect the serum level of miR‐195‐5p. Smoking history was defined as smoking ≥ 3 cigarettes per day for over 1 year and history of alcohol consumption was defined as daily alcohol consumption > 100 g for over 1 year.

### 2.4 Follow‐up

Patients were followed up for 3 months, with an interval of 1 week, starting from the time of enrollment. Patient outcomes were assessed and recorded using the Glasgow Outcome Scale (GOS). The scale consists of five grades: death (D), vegetative survival (VS), severe disability (SD), mild disability (MD), and good recovery (GR), among which GOS score 4–5 indicated good prognosis, and GOS score 1–3 indicated poor prognosis. The grade was jointly assessed by two attending neurosurgeons in The First Affiliated Hospital of Shantou University Medical College.

### 2.5 ELISA

The levels of IL‐6 (EK‐H10352, EK‐Bioscience, Shanghai, China) and ET‐1(EK‐H11291, EK‐Bioscience) in the serum were detected following the instructions of ELISA kits. Absorbance was measured at 450 nm with a microplate reader (Omron, Japan).

### 2.6 RT‐qPCR

RT‐qPCR was employed to measure the miR‐195‐5p and PDGF levels in the serum of all enrolled study population. The whole blood samples were collected in a 1.5 mL centrifuge tube without RNA enzyme and stored in a refrigerator at −80°C. RNA concentration was determined within 1 week. The blood samples were centrifuged at 3000 rpm for 20 min to extract the supernatant. TRIzol reagent (Thermo Fisher, MA, USA) was utilized to extract total RNA of the samples. Total RNA was isolated using mirVana PARIS kits and cDNA was synthesized by reverse transcription using PrimeScript RT Reagent kits (TaKaRa, Otsu, Shiga, Japan). The concentration and purity of extracted RNA was determined using ultrafine spectrophotometer (NanoDrop One, Thermo Fisher). ChamQ™ SYBR qRT‐PCR MasterMix (Vazyme Biotech, Nanjing, China) was adopted for the RT‐qPCR under reaction conditions of 95°C for 30 s, and 35 cycles of 95°C for 10 s and 60°C for 10 s. The relative levels of miR‐195‐5p and PDGF standardized by internal reference U6 and β‐actin were calculated by the 2^−ΔΔCt^ method (Livak and Schmittgen, 2001). RT‐qPCR primers were synthesized by Sangon Biotech (Shanghai, China) and the primer sequences are shown in Table 1.

**TABLE 1:** Primer sequence of RT‐qPCR

Gene	Forward 5′−3′	Reverse 5′−3′
miR‐195‐5p	AGGCGGCGCCCAGGCA	AGTGCAGGGTCCGAGGTATT
U6	GGAGACACGCAAACGGAAG	AGTGCAGGGTCCGAGGTATT
PDGF	GCTTTGGCTTTGGCTATCAG	CACTCTGTCTGCCCTTCTCC
β‐actin	CTGTGGCATCCACGAAACTA	GAGCCAGAGCAGTGATCTCC

### 2.7 Statistical analysis

SPSS21.0 statistical software (IBM Corp. Armonk, NY, USA) and GraphPad Prism 6.0 software (GraphPad Software Inc., San Diego, CA, USA) were used for statistical analyses and plotting. Shapiro‐Wilk test was used for checking the normal distribution. Measurement data of normal distribution were expressed as mean ± standard deviation, and the *t*‐test was used for data comparison between groups. Counting data were expressed as the number of cases and percentage, and chi‐square test was used for data comparison between groups. Receiver operating characteristic (ROC) curve was utilized to evaluate the diagnostic efficacy of parameters and obtain cut‐off values. The correlation between miR‐195‐5p and the serum levels of PDGF, IL‐6, and ET‐1 in patients with SAH + CVS was analyzed using the Pearson's method. Logistic regression was used to ascertain whether miR‐195‐5p was an independent risk factor for SAH + CVS. Kaplan‐Meier method was utilized to analyze the influence of miR‐195‐5p levels on the incidence of adverse prognosis, and COX regression model was employed to analyze the relationship between miR‐195‐5p and adverse prognosis. Differences were considered statistically significant at *p* < .05.

## 3 RESULTS

### 3.1 Comparative analysis of clinical data of the enrolled population

There was no statistical difference in age, gender, and history of smoking and alcohol consumption among patients with simple SAH, patients with SAH + CVS, and healthy subjects. There was no significant difference in the proportion of hypertension history between patients with SAH and patients with SAH + CVS (all *p* > .05)； the blood glucose, blood sodium fluctuation, serum lactic acid, IL‐6, ET‐1, and PDGF were raised in patients with SAH and patients with SAH + CVS compared with healthy subjects, and upregulated in patients with SAH + CVS compared with patients with SAH (all *p* < .05, Table 2).

**TABLE 2:** Comparison of clinical baseline features

	Control	SAH	SAH + CVS	*X* ^2^	*p*
Number of patients (cases)	60	62	58	–	–
Age (years)	52.28 ± 12.76	53.32 ± 13.80	54.73 ± 12.18	–	.5889
Gender (male/female)	33/27	29/33	35/23	2.266	.3221
Smoking history (yes/no)	28/32	36/26	37/21	3.659	.1605
Drinking history (yes/no)	26/34	32/30	38/20	5.944	.0512
Hypertension (yes/no)	–	25/37	18/40	1.124	.2890
Blood glucose (mmol/L)	4.94 ± 0.83	5.73 ± 0.96	6.17 ± 1.37		<.0001
Blood sodium fluctuation (mmol/L)	3.65 ± 2.13	5.36 ± 2.60	6.50 ± 2.87	–	<.0001
Serum lactic acid (mmol/L)	1.12 ± 0.36	1.51 ± 0.79	1.84 ± 0.97	–	<.0001
IL‐6 (μg/L)	6.37 ± 2.04	11.44 ± 5.82^a^	17.44 ± 9.73^a^, ^b^	–	<.0001
ET‐1 (pg/mL)	30.64 ± 3.91	47.62 ± 8.10^a^	55.26 ± 10.92^a^, ^b^	–	<.0001
PDGF (RPKM)	1.04 ± 0.32	1.42 ± 0.29	1.79 ± 0.41	–	<.0001

*Note*: Counting data were represented as the number of cases, and the chi‐square test was used for data comparison between groups; measurement data were expressed in quartiles, and data comparison between groups was performed using Mann–Whitney *U* test.

^a^ Significant difference compared with the data of the control group.

^b^ Significant difference compared with the data of the SAH group.

IL‐6: interleukin‐6; ET‐1: endothelin‐1; PDGF: platelet‐derived growth factor; RPKM: reads per kilobase per million mapped reads.

### 3.2 Downregulation of miR‐195‐5p in the serum of patients with SAH + CVS has high clinical diagnostic efficacy

**FIGURE 1:**
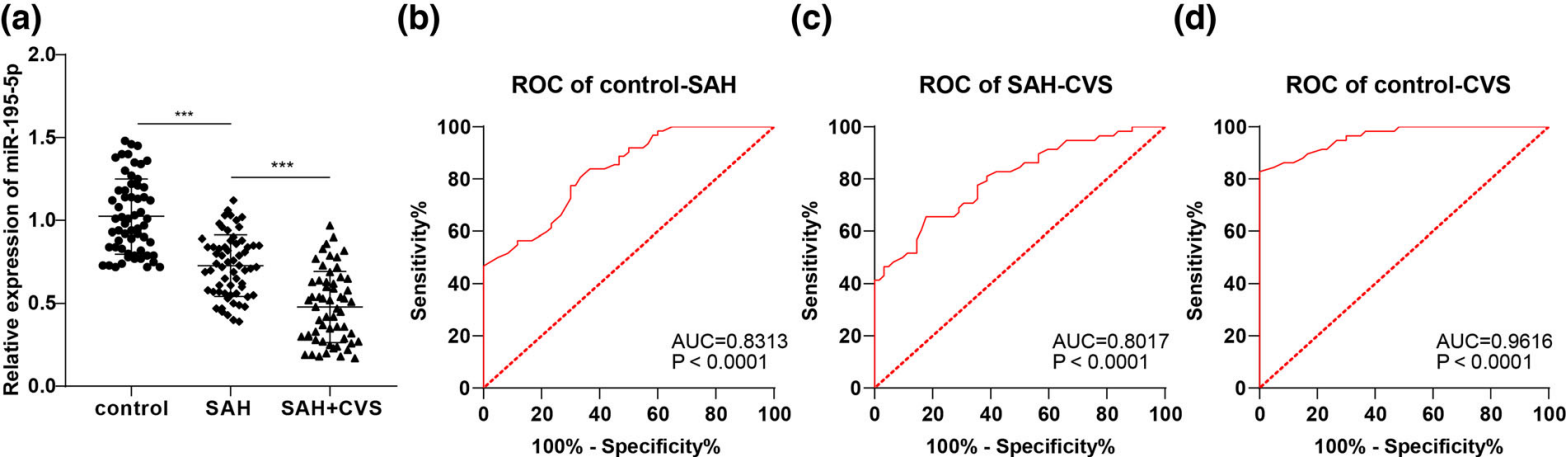
ROC curve of miR‐195‐5p levels in SAH and SHA + CVS diagnosis. (a) Serum level of miR‐195‐5p wa detected by RT‐qPCR. (b) ROC curve was used to analyze the diagnostic efficacy of serum miR‐195‐5p level on SAH. (c) ROC curve was adopted to analyze the diagnostic efficacy of serum miR‐195‐5p level in distinguishing SAH and SHA + CVS. (d) ROC curve was used to analyze the diagnostic efficacy of serum miR‐195‐5p level on SHA + CVS. Data were expressed as mean ± standard deviation. Data comparison between groups in (a) was analyzed by one‐way analysis of variance; ROC analysis was used for data comparison between groups in (b)–(d). ***p < .001

miR‐195‐5p expression in the serum of all subjects was compared by RT‐qPCR. The results manifested that miR‐195‐5p was 0.73 ± 0.18 in the serum of patients in the SAH group and 0.48 ± 0.21 in the SAH + CVS group, which was significantly lower than the control group (1.02 ± 0.23), and miR‐195‐5p in the SAH + CVS group was further less than that in the SAH group (all *p* < .05, Figure 1a).

Considering the abnormal serum expression of miR‐195‐5p in SAH patients and SAH + CVS patients, we further plotted the ROC curve for distinguishing subjects with different severity of disease based on miR‐195‐5p expression. MedCalc‐Comparison of ROC curves was used to compare the diagnostic performance of the control group, SAH group, and SAH + CVS group. The area under the curve (AUC) for identifying healthy subjects and SAH patients was 0.8313 and the cut‐off value was 0.8650, with 77.42% sensitivity and 70% specificity (Figure 1b); the AUC for distinguishing SAH patients and SAH + CVS patients was 0.8017 and the cut‐off value was 0.5450, with 65.52% sensitivity and 82.26% specificity (Figure 1c); the miR‐195‐5p level was adopted to distinguish healthy subjects from SAH + CVS patients with an AUC of 0.9616, cut‐off value of 0.7050, 82.76% sensitivity, and 100% specificity (Figure 1d). Overall, miR‐195‐5p serum level < 0.8650 can assist the diagnosis of SAH and miR‐195‐5p serum level < 0.5450 can assist the diagnosis of CVS after SAH.

### 3.3 Correlation analysis between miR‐195‐5p serum level and clinical indicators in the SAH and SAH + CVS group

**FIGURE 2:**
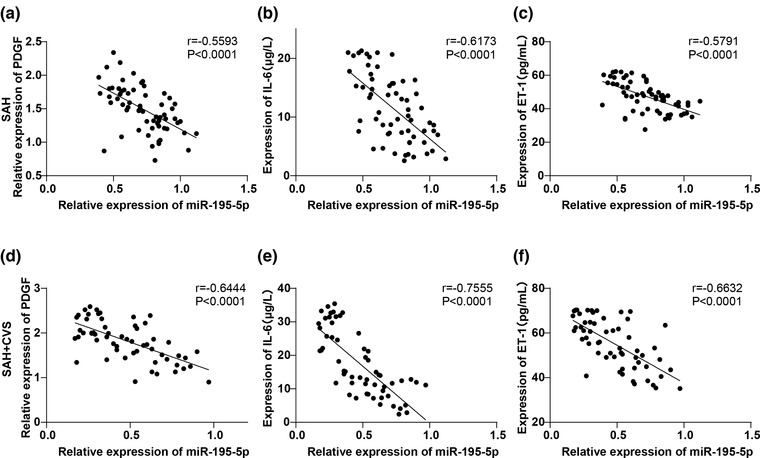
Correlation analysis of miR‐195‐5p with serum levels of PDGF, IL‐6 and ET‐1. (a) Correlation between the serum miR‐195‐5p level and PDGF in patients of the SAH group was analyzed using the Pearson's method. (b) Correlation between serum miR‐195‐5p level and IL‐6 of patients in the SAH group was analyzed using the Pearson's method. (c) Pearson's method was used to analyze the correlation between serum miR‐195‐5p level and ET‐1 of patients in the SAH group. (d) Pearson's method was used to analyze the correlation between serum miR‐195‐5p level and PDGF of patients in the SAH + CVS group. (e) Correlation between serum miR‐195‐5p level and IL‐6 of patients in the SAH + CVS group was analyzed by Pearson's method. (f) Pearson's method was used to analyze the correlation between serum miR‐195‐5p level and ET‐1 in the SAH + CVS group

Some serum indicators of SAH patients have been identified as reliable tools for diagnosis and treatment, including PDGF, IL‐6, and ET‐1 (Chaudhry et al., 2017; Ghali et al., 2018; Thampatty et al., 2011). Therefore, we further analyzed the correlation between miR‐195‐5p serum level and PDGF, IL‐6, and ET‐1 levels in the SAH group and SAH + CVS group by Pearson's analysis. It was discovered that miR‐195‐5p expression was negatively correlated with PDGF, IL‐6, and ET‐1 (Figure 2).

### 3.4 Independent correlation analysis between serum level of miR‐195‐5p and SAH + CVS

To figure out whether miR‐195‐5p is independently associated with SAH + CVS, we evaluated the independent risk factors affecting SAH + CVS by Logistic analysis. We took the prevalence of SAH + CVS as the dependent variable, all the indicators in Table 2 with the *p* value < .05 including blood glucose, blood sodium fluctuation, serum lactic acid, IL‐6, ET‐1, PDGF, and miR‐195‐5p as independent variables were included in the Logistic regression analysis model. After excluding the factors of blood glucose, blood sodium fluctuation, serum lactic acid, IL‐6, ET‐1, and PDGF, the miR‐195‐5p level was an independent correlation factor for the occurrence of SAH + CVS. Meanwhile, serum ET‐1 and blood glucose levels were also independently correlated with SAH + CVS (*p* < .05, Table 3).

**TABLE 3:** Logistic regression was used to analyze the independent correlation of miR‐195‐5p with SAH + CVS

Independent variables	*B* value	*p* Value	OR value	95% CI
miR	−8.069	.009	0.000	0.000–0.138
IL‐6	0.243	.103	0.952	0.952–1.706
ET‐1	0.467	.002	1.596	1.195–2.130
PDGF	0.532	.702	1.702	0.111–26.015
Blood glucose	1.606	.037	4.982	1.100–22.569
Blood sodium fluctuation	0.337	.356	1.401	0.964–2.035
Serum lactic acid	0.728	.853	2.072	0.442–9.721

### 3.5 Low expression of miR‐195‐5p predicted poor prognosis in patients with SAH + CVS

**FIGURE 3:**
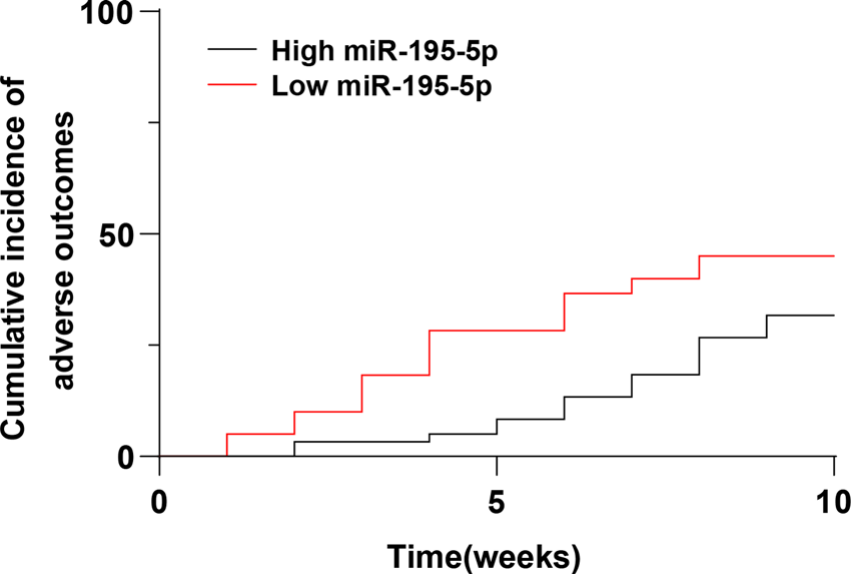
The Kaplan‐Meier method was used to analyze the effect of miR‐195‐5p levels on the prognosis of patients with SAH + CVS

All patients were divided into high‐expression (High miR) and low‐expression (Low miR) groups according to the median value of miR‐195‐5p level, and the incidence of adverse prognosis was compared. The results indicated that the incidence of poor prognosis in the low‐expression group was 45%, which was higher than that in the high‐expression group (31.67%), and there was a difference in the prognosis between the two groups (*X* ^2^ = 2.256, *p* = .1331) (Table 4). Moreover, Kaplan‐Meier analysis demonstrated that the curve of the low‐expression group shifted to the left (*p* = .0552, Figure 3), indicating that in the same follow‐up period, the cumulative incidence of poor prognosis was higher in the low‐expression group.

**TABLE 4:** Incidence of adverse outcomes

Group	High miR‐195‐5p (*N* = 60)	Low miR‐195‐5p (*N* = 60)	*X* ^2^	*p*
Dead (D)	2	5	0.6723	.7145
Vegetable survival (VS)	3	3		
Severe disability (SD)	14	19		
Mild disability (MD)	23	18	0.0178	.8938
Good recovery (GR)	18	15		
Total	19/41 (31.67%)	27/33 (45%)	2.256	.1331

### 3.6 Independent correlation analysis of miR‐195‐5p serum level and adverse prognosis of SAH + CVS

Further more, we analyzed whether miR‐195‐5p was independently correlated with the adverse prognosis of SAH + CVS. We took adverse prognosis as the dependent variable and all indicators in Table 2 with the *p* value < .05 including blood glucose, blood sodium fluctuation, serum lactic acid, IL‐6, ET‐1, PDGF, and miR‐195‐5p were included as independent variables in the multivariate COX regression analysis model. After excluding the factors of blood glucose, blood sodium fluctuation, serum lactic acid, IL‐6, ET‐1, and PDGF, the risk of adverse prognosis was increased by 0.052 times for every 1 unit enhance of miR‐195‐5p (*p* = .030, HR = 0.052, 95% Cl: 0.001‐0.746) (Table 5).

**TABLE 5:** COX regression model was used to analyze the independent correlation between miR‐195‐5p and the adverse prognosis of SAH + CVS

Independent variables	*B* value	*p* Value	HR value	95% CI
miR‐195‐5p	−2.964	.030	0.052	0.001–0.746
IL‐6	−0.003	.921	0.997	0.949–1.048
ET‐1	−0.010	.641	0.990	0.951–1.032
PDGF	−0.247	.617	0.781	0.297–2.056
Blood glucose	−0.009	.943	0.991	0.780–1.260
Blood sodium fluctuation	0.022	.689	1.022	0.918–1.138
Serum lactic acid	−0.025	.880	0.976	0.707–1.347

## 4 DISCUSSION

SAH is a serious neurological disease caused by cerebral hemorrhage entering the surrounding space of the brain, which occurs when the brain is anoxic due to various factors, especially blood supply interruption or aneurysm rupture (Cui et al., 2014). In addition, SAH causes bloody cerebrospinal fluid (CSF), while the bloody or highly proteinous CSF may lead to neural degeneration (Aydin et al., 2019). More importantly, CVS and increased intracranial pressure may be the main causes of neuron death after SAH (Celiker et al., 2019). As a major complication of SAH, the incidence of CVS is 30%−70% (Deng et al., 2021). One of the recognized indications of CVS is the presence of blood in subarachnoid space and the effect of hemoglobin degradation products on cerebral vessels (Kanat and Aydin, 1999). Nevertheless, so far, the occurrence of CVS has not been accurately anticipated (Liu et al., 2018). miRNAs perform a key role in neurodevelopment, neuroplasticity, and other neurobiological processes and diseases, and their abnormal regulation is associated with a range of neurological disorders (Lopes et al., 2018; Pulcrano‐Nicolas et al., 2018). Our findings demonstrated that the poor expression of miR‐195‐5p can assist the diagnosis of CVS following SAH and forecast adverse outcomes.

We first selected 120 patients with spontaneous SAH and allocated them into SAH group (*N* = 62) and SAH + CVS group (*N* = 58), and 60 healthy subjects were selected as controls. miR‐195‐5p, a member of the miR‐15 family, is a known tumor suppressor that is downregulated in several cancers (Giordano et al., 2019). Besides, miR‐195‐5p was found to be downregulated in ischemic/reperfusion (I/R) injury (Ren et al., 2021). In our study, serum miR‐195‐5p levels were reduced in SAH patients and further lowered in SAH + CVS patients. Similarly, miR‐195* levels were significantly decreased in arteriovenous malformations, the most common cause of nontraumatic intracerebral hemorrhage (Huang et al., 2017). Based on this, we further comprehensively compared the differences in diagnostic efficacy of miR‐195‐5p. The final data supported that serum miR‐195‐5p level < 0.8650 can assist the diagnosis of SAH; serum miR‐195‐5p level < 0.5450 can help identify SAH and CVS + SAH; serum miR‐195‐5p level < 0.7050 can identify healthy controls and CVS + SAH. The miRNA spectrum of cerebrospinal fluid samples in patients with CVS may be different from those without CVS (Stylli et al., 2017). The diagnostic value of other miRNAs has been reported. For instance, serum miR‐502‐5p and miR‐1297 levels were distinctly elevated in SAH patients in terms of severity, which could distinguish SAH patients from healthy subjects (Lai et al., 2017). There is evidence to suggest the therapeutic effect of miR‐195‐5p on SAH‐induced CVS in experimental SAH rats (Tsai et al., 2021). As a whole, our findings first highlighted that the low serum expression of miR‐195‐5p has high clinical diagnostic efficacy in patients with CVS following SAH.

Clinical indicators routinely monitored in SAH patients included glucose and sodium values, blood pressure, and serum lactic acid (Poblete et al., 2018; Roederer et al., 2014). As an inflammatory factor, IL‐6 is an important marker of SAH and subsequent development of CVS (Lucke‐Wold et al., 2021). ET‐1 and PDGF have also been implicated in CVS after SAH (Bellapart et al., 2014; Cui et al., 2014; Liu et al., 2018). In our current study, blood glucose, blood sodium fluctuation, serum lactic acid, IL‐6, ET‐1, and PDGF were all raised in SAH patients and were further elevated in SAH + CVS patients. The anti‐inflammatory effects of miR‐195‐5p have been documented in I/R injury and pulmonary diseases (Li et al., 2020; Xu et al., 2020). Considering the downregulation of miR‐195‐5p in the serum of patients with CVS following SAH, we speculated that the miR‐195‐5p serum level in SAH patients and SAH + CVS patients might be correlated with the levels of PDGF, IL‐6, and ET‐1. As expected, the miR‐195‐5p serum level was negatively correlated with PDGF, IL‐6, and ET‐1. miR‐195‐5p can reduce IL‐6 level and the release of inflammatory factors, which is in good agreement with our conclusion above (Zhu et al., 2021).

Effective prevention or treatment of CVS could significantly improve the survival and quality of life for aneurysmal SAH patients (Przybycien‐Szymanska and Ashley, 2015). Designed miRNA panel is an effective predictor of delayed CVS risk and has strong applications in the clinical management of SAH patients (Lan et al., 2020; Wang et al., 2021). Circulating miR‐195‐5p may serve as a biomarker and therapeutic target for transient ischemic attack and acute ischemic stroke (Giordano et al., 2019). miR‐195‐5p ameliorates cerebral I/R injury by regulating the PTEN‐AKT pathway (Ren et al., 2021). Furthermore, we analyzed the independent correlation between miR‐195‐5p and SAH + CVS. Interestingly, after excluding other clinical indicators, miR‐195‐5p level was an independent correlation factor for the occurrence of SAH + CVS. The CVS index was statistically positively correlated with changes in IL‐6 and ET‐1 (Liu et al., 2020). After SAH, increased synthesis of ET‐1 triggers enhanced CVS (Wanderer et al., 2020). Patients with symptomatic vasospasm had significantly higher mean in‐hospital blood glucose (Badjatia et al., 2005). Consistently, our results revealed that serum ET‐1 and blood glucose levels were likewise independently correlated with SAH + CVS.

Moreover, our study discovered that the incidence of adverse outcomes was markedly higher in patients with low expression of miR‐195‐5p than those with high expression of miR‐195‐5p. Additionally, after excluding other clinical indicators, an increase in 1 unit of miR‐195‐5p was associated with a 0.052‐fold increase in the risk of adverse outcomes. It is noteworthy that miR‐195‐5p mimic treatment prevented SAH‐CVS in the basilar artery (Tsai et al., 2021). There are scarce studies on the correlation of miR‐195‐5p levels and SAH‐CVS. But some other miRNAs have been studied. For example, a sustained increase in miR‐15a levels after SAH may contribute to changes in vascular phenotypes, leading to the development of CVS (Kikkawa et al., 2017). Upregulation of miR‐9 in cerebrospinal fluid after SAH was associated with poor prognosis (Bache et al., 2020). Our findings first revealed that low expression of miR‐195‐5p predicted the poor prognosis of SAH + CVS patients.

This study is the first to explore the clinical diagnostic value of miR‐195‐5p expression in the serum of patients with CVS after SAH and its relationship with adverse outcomes. Our findings provide a new entry point for clinical condition judgment and adverse outcome prediction. However, the number of cases and events included was limited, and the duration of follow‐up was short. Moreover, only TCD was adopted to detect the hemodynamic changes of patients with CVS, while digital subtraction angiography (DSA), as the gold standard for the diagnosis of CVS, was not employed in this study. In future studies, we need to conduct multi‐center prospective studies to further expand the sample size, extend the follow‐up time, and increase the credibility of our results. Additionally, the level of ET‐1 in blood glucose and serum can be further investigated to study its predictive value for CVS following SAH. Besides, DSA will be further utilized to detect the hemodynamic changes of patients with CVS.

## ETHICAL STATEMENT

This study was approved by the Ethics Committee of The First Affiliated Hospital of Shantou University Medical College. Patients and their families were fully informed of the purpose of this study, and all voluntarily participated in this study and signed informed consent prior to sampling.

### PEER REVIEW

The peer review history for this article is available at https://publons.com/publon/10.1002/brb3.2766.

## AVAILABILITY OF DATA AND MATERIALS

The data that support the findings of this study are available from the corresponding author upon reasonable request.

## CONFLICT OF INTEREST

All authors declare that there is no conflict of interests in this study.

## FUNDING

This work was supported by Medical and Health Project of Shantou Science and Technology Program (No. 220519126490399).

## AUTHOR CONTRIBUTIONS

YL is responsible for ensuring the integrity of the whole study and the definition of the study concept, knowledge content, clinical study; SYY is responsible for study design, data analysis, and statistical analysis; XBZ is responsible for literature research and experimental research; RLL is responsible for manuscript writing and data collection; YL and SYY are responsible for manuscript editing and review. All authors read and approved the final manuscript.

## References

[brb32766-bib-0001] Aydin, M. D. , Kanat, A. , Yolas, C. , Soyalp, C. , Onen, M. R. , Yilmaz, I. , Karaavci, N. C. , Calik, M. , Baykal, O. , & Ramazanoglu, L. (2019). Spinal subarachnoid hemorrhage induced intractable miotic pupil. A Reminder of Ciliospinal Sympathetic Center Ischemia Based Miosis: An experimental study. Turkish Neurosurgery, 29(3), 434–439. 10.5137/1019-5149.JTN.24446-18.1 30984987

[brb32766-bib-0002] Bache, S. , Rasmussen, R. , Wolcott, Z. , Rossing, M. , Mogelvang, R. , Tolnai, D. , Hassager, C. , Forman, J. L. , Køber, L. , Nielsen, F. C. , Kimberly, W. T. , & Moller, K. (2020). Elevated miR‐9 in cerebrospinal fluid is associated with poor functional outcome after subarachnoid hemorrhage. Translational Stroke Research, 11(6), 1243–1252. 10.1007/s12975-020-00793-1 32248435

[brb32766-bib-0003] Badjatia, N. , Topcuoglu, M. A. , Buonanno, F. S. , Smith, E. E. , Nogueira, R. G. , Rordorf, G. A. , Carter, B. S. , Ogilvy, C. S. , & Singhal, A. B. (2005). Relationship between hyperglycemia and symptomatic vasospasm after subarachnoid hemorrhage. Critical Care Medicine, 33(7), 1603–1609. quiz 1623. 10.1097/01.ccm.0000168054.60538.2b 16003069

[brb32766-bib-0004] Bellapart, J. , Jones, L. , Bandeshe, H. , & Boots, R. (2014). Plasma endothelin‐1 as screening marker for cerebral vasospasm after subarachnoid hemorrhage. Neurocritical Care, 20(1), 77–83. 10.1007/s12028-013-9887-1 23921571

[brb32766-bib-0005] Bounds, K. R. , Chiasson, V. L. , Pan, L. J. , Gupta, S. , & Chatterjee, P. (2017). MicroRNAs: New players in the pathobiology of preeclampsia. Frontiers in Cardiovascular Medicine, 4(60), 10.3389/fcvm.2017.00060 PMC5622156 28993808

[brb32766-bib-0006] Celiker, M. , Kanat, A. , Aydin, M. D. , Ozdemir, D. , Aydin, N. , Yolas, C. , Calik, M. , & Peker, H. O. (2019). First emerging objective experimental evidence of hearing impairment following subarachnoid haemorrhage; Felix culpa, phonophobia, and elucidation of the role of trigeminal ganglion. International Journal of Neuroscience, 129(8), 794–800. 10.1080/00207454.2019.1569651 30636470

[brb32766-bib-0007] Chaudhry, S. R. , Stoffel‐Wagner, B. , Kinfe, T. M. , Guresir, E. , Vatter, H. , Dietrich, D. , Lamprecht, A. , & Muhammad, S. (2017). Elevated systemic IL‐6 levels in patients with aneurysmal subarachnoid hemorrhage is an unspecific marker for post‐SAH complications. International Journal of Molecular Sciences, 18(12), 10.3390/ijms18122580 PMC5751183 29194369

[brb32766-bib-0008] Cui, H. K. , Yan, R. F. , Ding, X. L. , Zhao, P. , Wu, Q. W. , Wang, H. P. , Qin, H. ‐X. , Tu, J. ‐F. , & Yang, R. M. (2014). Platelet‐derived growth factor‐beta expression in rabbit models of cerebral vasospasm following subarachnoid hemorrhage. Molecular Medicine Reports, 10(3), 1416–1422. 10.3892/mmr.2014.2350 24969827

[brb32766-bib-0009] Deng, X. , Liang, C. , Qian, L. , & Zhang, Q. (2021). miR‐24 targets HMOX1 to regulate inflammation and neurofunction in rats with cerebral vasospasm after subarachnoid hemorrhage. American Journal of Translational Research, 13(3), 1064–1074. 33841640 PMC8014398

[brb32766-bib-0010] Dubosh, N. M. , & Edlow, J. A. (2021). Diagnosis and initial emergency department management of subarachnoid hemorrhage. Emergency Medicine Clinics of North America, 39(1), 87–99. 10.1016/j.emc.2020.09.005 33218664

[brb32766-bib-0011] Etminan, N. , & Macdonald, R. L. (2021). Neurovascular disease, diagnosis, and therapy: Subarachnoid hemorrhage and cerebral vasospasm. Handbook of Clinical Neurology, 176, 135–169. 10.1016/B978-0-444-64034-5.00009-2 33272393

[brb32766-bib-0012] Ghali, M. G. Z. , Srinivasan, V. M. , Johnson, J. , Kan, P. , & Britz, G. (2018). Therapeutically targeting platelet‐derived growth factor‐mediated signaling underlying the pathogenesis of subarachnoid hemorrhage‐related vasospasm. Journal of Stroke and Cerebrovascular Diseases, 27(9), 2289–2295. 10.1016/j.jstrokecerebrovasdis.2018.02.017 30037648

[brb32766-bib-0013] Giordano, M. , Ciarambino, T. , D'Amico, M. , Trotta, M. C. , Di Sette, A. M. , Marfella, R. , Malatino, L. , Paolisso, G. , & Adinolfi, L. E. (2019). Circulating MiRNA‐195‐5p and ‐451a in transient and acute ischemic stroke patients in an emergency department. Journal of Clinical Medicine, 8(2). 10.3390/jcm8020130 PMC6406765 30678250

[brb32766-bib-0014] Gulec, I. , Sengelen, A. , Karagoz‐Guzey, F. , Onay‐Ucar, E. , Eren, B. , Vahabova, G. , Karacan, M. , & Ozcan, T. B. (2021). The calcimimetic R‐568 attenuates subarachnoid hemorrhage‐induced vasospasm through PI3K/Akt/eNOS signaling pathway in the rat model. Brain Research, 1765, 147508. 10.1016/j.brainres.2021.147508 33930376

[brb32766-bib-0015] Haruma, J. , Teshigawara, K. , Hishikawa, T. , Wang, D. , Liu, K. , Wake, H. , Mori, S. , Kohka Takahashi, H. , Sugiu, K. , Date, I. , & Nishibori, M. (2016). Anti‐high mobility group box‐1 (HMGB1) antibody attenuates delayed cerebral vasospasm and brain injury after subarachnoid hemorrhage in rats. Science Reports, 6, 37755. 10.1038/srep37755 PMC5121891 27883038

[brb32766-bib-0016] Huang, J. , Song, J. , Qu, M. , Wang, Y. , An, Q. , Song, Y. , Yan, W. , Wang, B. , Wang, X. , Zhang, S. , Chen, X. , Zhao, B. , Liu, P. , Xu, T. , Zhang, Z. , Greenberg, D. A. , Wang, Y. , Gao, P. , Zhu, W. , & Yang, G. Y. (2017). MicroRNA‐137 and microRNA‐195* inhibit vasculogenesis in brain arteriovenous malformations. Annals of Neurology, 82(3), 371–384. 10.1002/ana.25015 28802071

[brb32766-bib-0017] Kanat, A. , & Aydin, Y. (1999). Selection of cerebral aneurysms for treatment using Guglielmi detachable coils: The preliminary University of Illinois at Chicago experience. Neurosurgery, 45(3), 670–674. 10.1097/00006123-199909000-00054 10493392

[brb32766-bib-0018] Kikkawa, Y. , Ogura, T. , Nakajima, H. , Ikeda, T. , Takeda, R. , Neki, H. , Kohyama, S. , Yamane, F. , Kurogi, R. , Amano, T. , Nakamizo, A. , Mizoguchi, M. , & Kurita, H. (2017). Altered Expression of MicroRNA‐15a and Kruppel‐Like Factor 4 in Cerebrospinal Fluid and Plasma After Aneurysmal Subarachnoid Hemorrhage. World Neurosurgery, 108, 909–916. e903. 10.1016/j.wneu.2017.09.008 28893694

[brb32766-bib-0019] Lai, N. S. , Zhang, J. Q. , Qin, F. Y. , Sheng, B. , Fang, X. G. , & Li, Z. B. (2017). Serum microRNAs are non‐invasive biomarkers for the presence and progression of subarachnoid haemorrhage. Bioscience Reports, 37(1). 10.1042/BSR20160480 PMC5322746 28115593

[brb32766-bib-0020] Lan, S. , Zhou, L. , Wang, Y. , Fang, L. , Yang, L. , Zheng, S. , Zhou, X. H. , Tang, B. , Duan, J. , Wu, X. , Yang, C. , & Hong, T. (2020). miRNA profiling of circulating small extracellular vesicles from subarachnoid hemorrhage rats using next‐generation sequencing. Frontiers in Cellular Neuroscience, 14, 242. 10.3389/fncel.2020.00242 32903819 PMC7439219

[brb32766-bib-0021] Li, H. T. , Wang, J. , Li, S. F. , Cheng, L. , Tang, W. Z. , & Feng, Y. G. (2018). Upregulation of microRNA24 causes vasospasm following subarachnoid hemorrhage by suppressing the expression of endothelial nitric oxide synthase. Molecular Medicine Reports, 18(1), 1181–1187. 10.3892/mmr.2018.9050 29845232

[brb32766-bib-0022] Li, S. , Jiang, L. , Yang, Y. , Cao, J. , Zhang, Q. , Zhang, J. , Wang, R. , Deng, X. , & Li, Y. (2020). MiR‐195‐5p inhibits the development of chronic obstructive pulmonary disease via targeting siglec1. Human & Experimental Toxicology, 39(10), 1333–1344. 10.1177/0960327120920923 32351126

[brb32766-bib-0023] Liu, J. P. , Ye, Z. N. , Lv, S. Y. , Zhuang, Z. , Zhang, X. S. , Zhang, X. , Wu, W. , Mao, L. , Lu, Y. , Wu, L. ‐Y. , Fan, J. ‐M. , Tian, W. ‐J. , & Hang, C. H. (2018). The rise of soluble platelet‐derived growth factor receptor beta in CSF early after subarachnoid hemorrhage correlates with cerebral vasospasm. Neurological Sciences, 39(6), 1105–1111. 10.1007/s10072-018-3329-y 29637448

[brb32766-bib-0024] Liu, X. , Zhao, N. , Zeng, K. , Xiao, P. , Sheng, P. , Luo, X. , & Wang, Y. (2020). Effects of nimodipine combined with betahistine on CRP and other inflammatory cytokines and vascular endothelial function in patients with hypertensive cerebral vasospasm. Clinical Hemorheology and Microcirculation, 75(3), 279–289. 10.3233/CH-190589 32280080

[brb32766-bib-0025] Livak, K. J. , & Schmittgen, T. D. (2001). Analysis of relative gene expression data using real‐time quantitative PCR and the 2(‐Delta Delta C(T)) Method. Methods (San Diego, Calif.), 25(4), 402–408. 10.1006/meth.2001.1262 11846609

[brb32766-bib-0026] Lopes, K. P. , Vinasco‐Sandoval, T. , Vialle, R. A. , Paschoal, F. M. Jr. , Bastos, V. , Bor‐Seng‐Shu, E. , Aviz Bastos, V. A. P. , Bor‐Seng‐Shu, E. , Jacobsen Teixeira, M. , Sumi Yamada, E. , Pinto, P. , Ferreira Vidal, A. , Ribeiro‐Dos‐Santos, A. , Moreira, F. , Santos, S. , Homero Albuquerque Paschoal, E. , & Ribeiro‐Dos‐Santos, A. (2018). Global miRNA expression profile reveals novel molecular players in aneurysmal subarachnoid haemorrhage. Science Reports, 8(1), 8786. 10.1038/s41598-018-27078-w PMC5993784 29884860

[brb32766-bib-0027] Lucke‐Wold, B. , Hosaka, K. , Dodd, W. , Motwani, K. , Laurent, D. , Martinez, M. , & Hoh, B. (2021). Interleukin‐6: Important mediator of vasospasm following subarachnoid hemorrhage. Current Neurovascular Research, 18(3), 364–369. 10.2174/1567202618666211104122408 34736380 PMC10127255

[brb32766-bib-0028] Neifert, S. N. , Chapman, E. K. , Martini, M. L. , Shuman, W. H. , Schupper, A. J. , Oermann, E. K. , Mocco, J. , & Macdonald, R. L. (2021). Aneurysmal subarachnoid hemorrhage: The last decade. Translational Stroke Research, 12(3), 428–446. 10.1007/s12975-020-00867-0 33078345

[brb32766-bib-0029] Poblete, R. A. , Cen, S. Y. , Zheng, L. , & Emanuel, B. A. (2018). Serum lactic acid following aneurysmal subarachnoid hemorrhage is a marker of disease severity but is not associated with hospital outcomes. Frontiers in Neurology , 9(593). 10.3389/fneur.2018.00593 PMC6064931 30083130

[brb32766-bib-0030] Przybycien‐Szymanska, M. M. , & Ashley, W. W. Jr. (2015). Biomarker discovery in cerebral vasospasm after aneurysmal subarachnoid hemorrhage. Journal of Stroke and Cerebrovascular Diseases, 24(7), 1453–1464. 10.1016/j.jstrokecerebrovasdis.2015.03.047 25957908

[brb32766-bib-0031] Pulcrano‐Nicolas, A. S. , Proust, C. , Clarencon, F. , Jacquens, A. , Perret, C. , Roux, M. , Shotar, E. , Thibord, F. , Puybasset, L. , Garnier, S. , Degos, V. , & Tregouet, D. A. (2018). Whole‐blood miRNA sequencing profiling for vasospasm in patients with aneurysmal subarachnoid hemorrhage. Stroke; A Journal of Cerebral Circulation, 49(9), 2220–2223. 10.1161/STROKEAHA.118.021101 30354977

[brb32766-bib-0032] Ren, X. , Wang, Z. , & Guo, C. (2021). MiR‐195‐5p ameliorates cerebral ischemia‐reperfusion injury by regulating the PTEN‐AKT signaling pathway. Neuropsychiatric Disease and Treatment, 17, 1231–1242. 10.2147/NDT.S297975 33958865 PMC8093143

[brb32766-bib-0033] Roederer, A. , Holmes, J. H. , Smith, M. J. , Lee, I. , & Park, S. (2014). Prediction of significant vasospasm in aneurysmal subarachnoid hemorrhage using automated data. Neurocritical Care, 21(3), 444–450. 10.1007/s12028-014-9976-9 24715326

[brb32766-bib-0034] Saugstad, J. A. (2010). MicroRNAs as effectors of brain function with roles in ischemia and injury, neuroprotection, and neurodegeneration. Journal of Cerebral Blood Flow and Metabolism, 30(9), 1564–1576. 10.1038/jcbfm.2010.101 20606686 PMC2932764

[brb32766-bib-0035] Shang, F. , Zhao, H. , Cheng, W. , Qi, M. , Wang, N. , & Qu, X. (2021). Predictive value of the serum albumin level on admission in patients with spontaneous subarachnoid hemorrhage. Frontiers in Surgery, 8, 719226. 10.3389/fsurg.2021.719226 34765635 PMC8576111

[brb32766-bib-0036] Smits, A. , Kato, M. , Westermark, B. , Nister, M. , Heldin, C. H. , & Funa, K. (1991). Neurotrophic activity of platelet‐derived growth factor (PDGF): Rat neuronal cells possess functional PDGF beta‐type receptors and respond to PDGF. PNAS, 88(18), 8159–8163. 10.1073/pnas.88.18.8159 1654560 PMC52466

[brb32766-bib-0037] Stylli, S. S. , Adamides, A. A. , Koldej, R. M. , Luwor, R. B. , Ritchie, D. S. , Ziogas, J. , & Kaye, A. H. (2017). miRNA expression profiling of cerebrospinal fluid in patients with aneurysmal subarachnoid hemorrhage. Journal of Neurosurgery, 126(4), 1131–1139. 10.3171/2016.1.JNS151454 27128592

[brb32766-bib-0038] Thampatty, B. P. , Sherwood, P. R. , Gallek, M. J. , Crago, E. A. , Ren, D. , Hricik, A. J. , Kuo, C.‐W. J. , Klamerus, M. M. , Alexander , S. A. , Bender, C. M. , Hoffman, L. A. , Horowitz, M. B. , Kassam, A. B. , & Poloyac, S. M. (2011). Role of endothelin‐1 in human aneurysmal subarachnoid hemorrhage: Associations with vasospasm and delayed cerebral ischemia. Neurocritical Care, 15(1), 19–27. 10.1007/s12028-011-9508-9 21286855 PMC3134137

[brb32766-bib-0039] Tsai, T. H. , Chang, C. H. , Lin, S. H. , Su, Y. F. , Tsai, Y. C. , Yang, S. F. , & Lin, C. L. (2021). Therapeutic effect of and mechanisms underlying the effect of miR‐195‐5p on subarachnoid hemorrhage‐induced vasospasm and brain injury in rats. PeerJ, 9, e11395. 10.7717/peerj.11395 34221706 PMC8231314

[brb32766-bib-0040] van Heyningen, P. , Calver, A. R. , & Richardson, W. D. (2001). Control of progenitor cell number by mitogen supply and demand. Current Biology, 11(4), 232–241. 10.1016/s0960-9822(01)00075-6 11250151

[brb32766-bib-0041] Wanderer, S. , Gruter, B. E. , Strange, F. , Sivanrupan, S. , Di Santo, S. , Widmer, H. R. , Fandino, J. , Marbacher, S. , & Andereggen, L. (2020). The role of sartans in the treatment of stroke and subarachnoid hemorrhage: A narrative review of preclinical and clinical studies. Brain Sciences, 10(3). 10.3390/brainsci10030153 PMC7139942 32156050

[brb32766-bib-0042] Wang, W. X. , Springer, J. E. , Xie, K. , Fardo, D. W. , & Hatton, K. W. (2021). A highly predictive microrna panel for determining delayed cerebral vasospasm risk following aneurysmal subarachnoid hemorrhage. Frontiers in Molecular Biosciences, 8, 657258. 10.3389/fmolb.2021.657258 34055880 PMC8163224

[brb32766-bib-0043] Xu, L. , Wu, J. , Liu, Y. , Chen, G. , Ma, C. , & Zhang, H. (2021). Peroxisome proliferatoractivated receptor beta/delta regulates cerebral vasospasm after subarachnoid hemorrhage via modulating vascular smooth muscle cells phenotypic conversion. Molecular Medicine Reports, 24(6). 10.3892/mmr.2021.12500 PMC8548938 34664679

[brb32766-bib-0044] Xu, Y. , Jiang, W. , Zhong, L. , Li, H. , Bai, L. , Chen, X. , Lin, Y. , & Zheng, D. (2020). miR‐195‐5p alleviates acute kidney injury through repression of inflammation and oxidative stress by targeting vascular endothelial growth factor A. Aging (Albany NY), 12(11), 10235–10245. 10.18632/aging.103160 32492657 PMC7346085

[brb32766-bib-0045] Zhang, Z. W. , Yanamoto, H. , Nagata, I. , Miyamoto, S. , Nakajo, Y. , Xue, J. H. , Iihara, K. , & Kikuchi, H. (2010). Platelet‐derived growth factor‐induced severe and chronic vasoconstriction of cerebral arteries: Proposed growth factor explanation of cerebral vasospasm. Neurosurgery, 66(4), 728–735. discussion 735. 10.1227/01.NEU.0000366111.08024.26 20305494

[brb32766-bib-0046] Zhu, L. L. , Wang, H. Y. , & Tang, T. (2021). Effects of miR‐195 on diabetic nephropathy rats through targeting TLR4 and blocking NF‐kappaB pathway. European Review for Medical and Pharmacological Sciences, 25(3), 1522–1529. 10.26355/eurrev_202102_24860 33629321

